# Multivariate chemometric profiling of Himalayan *Ganoderma* isolates: linking host and altitude to nutritional, antioxidant, and metabolomic diversity

**DOI:** 10.1039/d6ra04253h

**Published:** 2026-07-27

**Authors:** Sonali Khanal, Pankaj Kumar, Purnima Sharma, Vinay Chauhan, Rachna Verma, Ashwani Tapwal, Dinesh Kumar, Vinod Kumar

**Affiliations:** a School of Bioengineering and Food Technology, Shoolini University of Biotechnology and Management Sciences Solan 173229 India dineshkumar@shooliniuniversity.com; b School of Advanced Chemical Sciences, Shoolini University Solan 173229 India; c ICFRE-Himalayan Forest Research Institute Shimla 171013 India; d School of Biological and Environmental Science, Shoolini University of Biotechnology and Management Sciences Solan 173229 India; e Faculty of Materials Science & Technology, VSB – Technical University of Ostrava Ostrava-Poruba 708 00 Czech Republic; f Magan Centre for Applied Mycology, Cranfield University Cranfield MK43 0AL UK vinod.kumar@cranfield.ac.uk

## Abstract

Five Himalayan *Ganoderma* isolates (*G. leucocontextum*: GL11, GL13, GL17; *G. lucidum*: GL20, GL24) were analysed for nutritional composition, mineral content, antioxidant activity, and metabolomic fingerprints. Proximate analysis revealed significant host-dependent variation, with protein ranging from 15.26 ± 0.14% to 18.49 ± 0.27%. Principal component analysis (PCA) of mineral data explained 85.78% of variance, with *Quercus*-associated high-altitude isolates clustering together; Pearson correlation highlighted strong Ca–Sr and Na–Zn associations and negative Mn–Na and Fe–Ca interactions. Antioxidant assays showed species-level divergence: *G. leucocontextum* isolates exhibited lower phenolics (1.31 ± 0.01–1.78 ± 0.01 mg GAE per g) and terpenoids (2.37 ± 0.005–3.60 ± 0.003 mg UA per g) with weaker FRAP (76.66 ± 0.001–111.42 ± 0.003 µmol g^−1^), while *G. lucidum* isolates showed higher phenolics (2.83 ± 0.02–4.54 ± 0.04 mg GAE per g), terpenoids (5.82 ± 0.009–6. 36 ± 0.01 mg UA per g) stronger FRAP (133.33 ± 0.006–162.38 ± 0.02 µmol g^−1^), and lower IC_50_ values (208.09 ± 13.23–211.28 ± 3.35). HCA validated the species-specific separation, categorizing *G. leucocontextum* strains in one group and *G. lucidum* in another. Metabolomics using GC-MS revealed 179 metabolites, with PCA explaining 85.16% of the variability. Primary separation was performed by lipid esters such as ethyl-hexadecanoate, while secondary differentiation was performed by aromatic compounds such as 1*H*-indene, 1-methylene-, and aniline derivatives. Lipid esters established wide differentiation, while aromatic compounds were used for subtle chemical fingerprinting. Both PCA and HCA demonstrate that ecological context plays an important role in shaping the biochemical profile of *Ganoderma*.

## Abstract 2

Five Himalayan *Ganoderma* isolates (*G. leucocontextum*: GL11, GL13, GL17; *G. lucidum*: GL20, GL24) were analysed for nutritional composition, mineral content, antioxidant activity, and metabolomic fingerprints.

## Graphical abstract

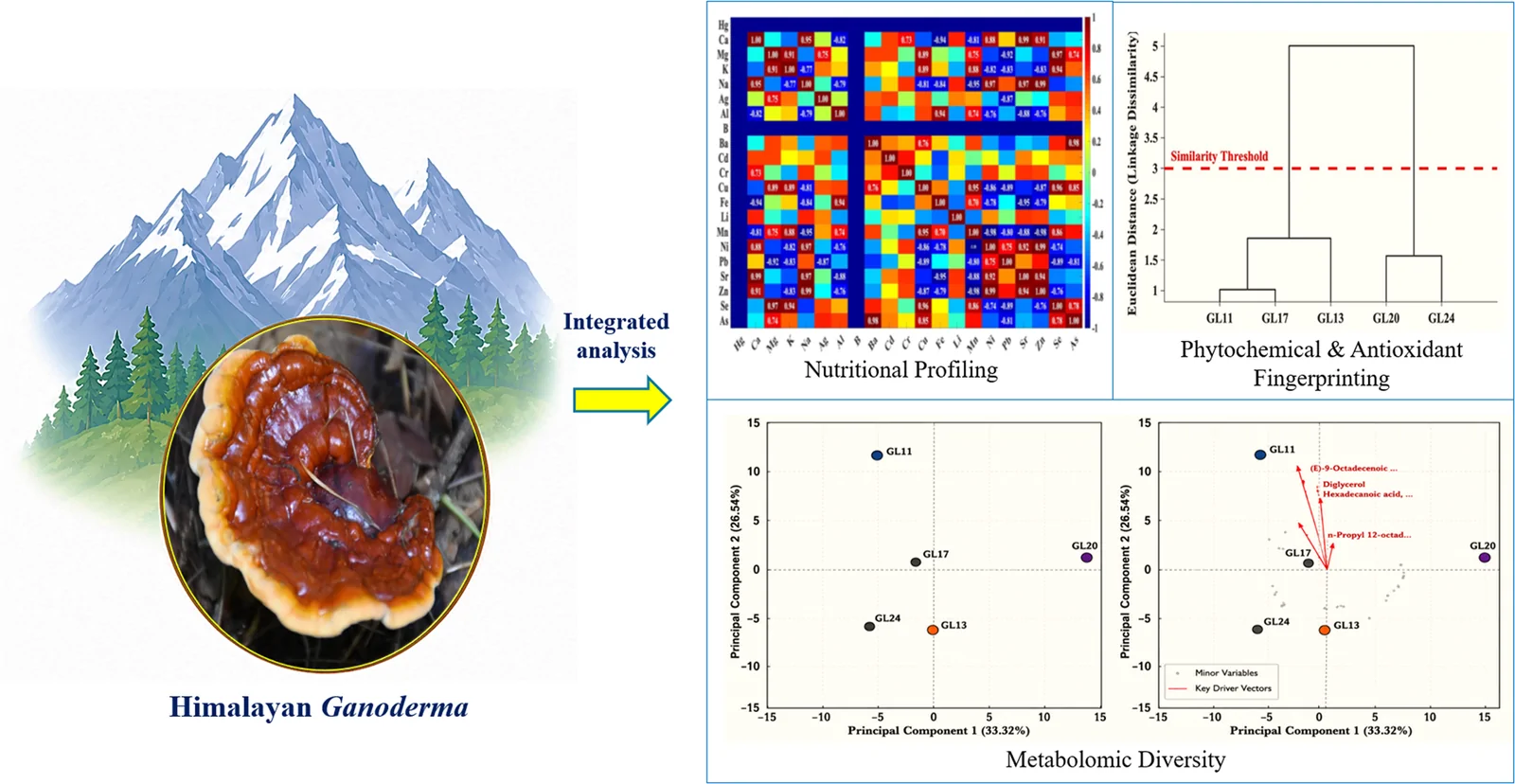

## 1. Introduction

The various *Ganoderma* genera, especially those which occur in the ecological niches of the Himachal Himalaya, are revered as “immortal mushrooms” owing to their wide range of pharmaceutical qualities, such as powerful antioxidants, immunoregulatory, and anti-inflammatory activity.^1^ The medicinal importance of these mushrooms has worldwide acceptance; however, their biological activity cannot be separated from the chemical composition.^2^ In particular, the antioxidant capability, which is regulated by secondary compounds such as phenolic and terpenoids, plays a fundamental role in defining the pharmaceutical quality of *Ganoderma*. However, the chemical dynamics within *Ganoderma* are highly variable, showing marked variations depending on the host and elevation level.^3,4^

This natural metabolic variation is, in fact, one of the challenges associated with the industrialization and standardization of *Ganoderma*. Variations in the types of host trees in the Himachal Himalaya have been found to cause variations in their proximate composition, mineral content, phenol concentration, and antioxidant activity, even though morphological characterization may not be able to detect chemical differences, which are important in determining high-potency chemotypes.^5^ Variations in the host tree types in the Himachal Himalaya have been found to cause variations in their proximate composition, mineral content, phenol concentration, and antioxidant activity, even though morphological characterization may not be able to detect chemical differences, which are crucial in identifying high-quality types.^6^ The chemical complexity and quality assurance of *Ganoderma* were studied by various researchers. The nutritional and pharmacological properties of *G. lucidum* and *Pleurotus ostreatus* were compared using GC-MS and molecular techniques, leading to the identification of seventy bioactive compounds with antimicrobial, antioxidant, and anticancer effects.^7^ In chemometrics for ganoderic acids among different cultivars, the relationship between chemical fingerprinting and anti-proliferation was determined, indicating triterpenoids as bioactive indicators.^8^ The elemental composition of ten *Ganoderma* species was characterized through macro-, micro-, and trace elements, with principal component analysis able to differentiate between cultivated and wild samples.^9^ In recent years, isotope ratio mass spectrometry and multivariate modeling were employed for accurate classification of *G. lucidum* based on development stage, cultivar, and geographical distribution.^10^ Although these works have shown that chemical profiling and multivariate analysis techniques hold great potential for *Ganoderma* species identification and evaluation of their biological activities, almost all have considered only one aspect. Very few studies have analyzed different proximate, mineral, antioxidant, and metabolomic data sets together or associated these with ecological factors like host tree and altitude. To address this ‘standardization gap,’ the current work employs an integrated analytical method. The nutritional differences between the isolates were statistically confirmed with ANOVA for proximate composition. Principal Component Analysis (PCA) and Pearson's correlation coefficient were used to identify variance structure and positive and negative correlations between various mineral components during mineral profiling, which links mineral abundance to ecological stratification. Hierarchical Cluster Analysis (HCA) and PCA were used to identify the isolates based on their scavenging and reduction abilities, revealing species-specific differences between *G. leucocontextum* and *G. lucidum*. Metabolomics analysis with GC-MS was performed in conjunction with PCA, yielding high-resolution metabolite fingerprints and the identification of 179 compounds.

Thus, the main objective of this study is to find out how the ecological environment, such as host plant and altitude, affects the nutritional content, antioxidant capacity, and metabolic profiles of *Ganoderma* in the Himalayas. Using proximate composition, chemical elements analysis, antioxidant analysis, and metabolomics under an integrated statistical approach, this paper seeks to provide chemotaxonomic tools for the identification, evaluation, and medicinal use of these precious fungi.

## 2. Materials and methods

### 2.1. Reagents and chemicals

Reagents and chemicals were of analytical grade, assuring the premium quality requirements for precise and dependable results. DPPH (2,2′-diphenyl-1-picrylhydrazyl), Folin–Ciocalteu reagent, ethanol, methanol, glacial acetic acid, gallic acid, ascorbic acid, and sodium carbonate were purchased from HiMedia Laboratories, India. Perchloric acid (70%) was purchased from Simson, India. TPTZ (2,4,6-tripyridyl-*s*-triazine, Fluka Chemicals, Switzerland). Ursolic acid was purchased from Med Chem Express (HY-N0140).

### 2.2. Collection of the samples and preparation

**Fig. 1:**
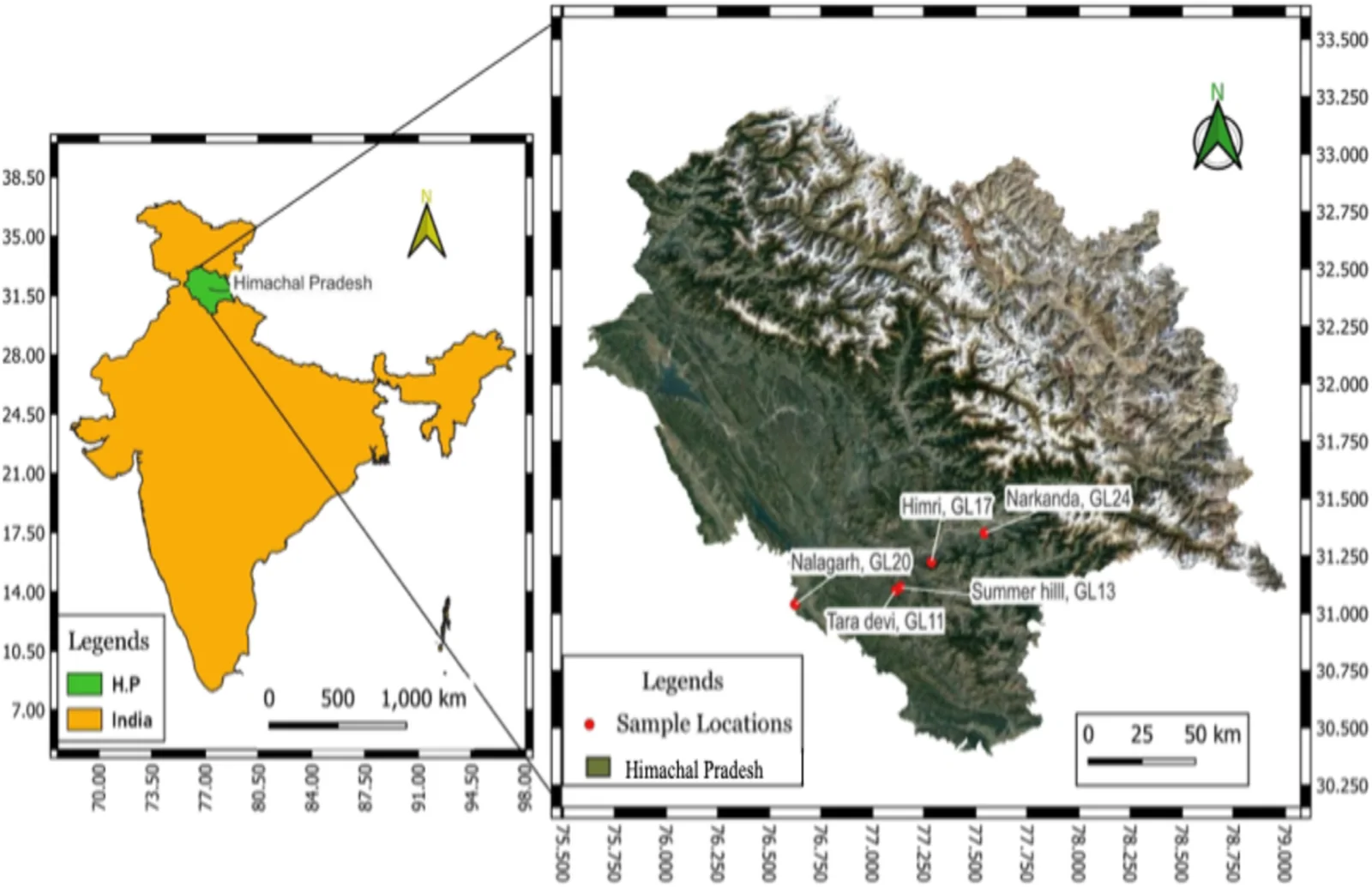
Geographic distribution of sampling sites in Himachal Pradesh, India.

Five distinct *Ganoderma* isolates were collected from various host species in different agro-climatic zones of Himachal Pradesh, India. The cohort comprised two species: *Ganoderma lucidum* (GL20 and GL24) and *Ganoderma leucocontextum* (GL11, GL13, and GL17). The geographic distribution of these sampling sites across the Himalayan terrain is illustrated in Fig. 1, with specific topographic metadata and host associations detailed in Table 1. The fruiting bodies were dried in a controlled manner at 45 °C until they reached a consistent mass as reported in the previous study^11^ and were ground to a uniform size using a grinder (Glen Mixer, Grinder). The uniformly sized dry powder sample was subsequently sieved using a 100 mm mesh and stored at 4 °C to maintain its integrity and pharmacological characteristics before extraction. All samples were analyzed for all tests, and all assays were conducted in triplicate.

**Table 1:** Hosts and sites of collection of *Ganoderma* in Himachal Pradesh, India

Isolates no.	Host tree	Host types	Site of collection	GPS coordinates	Elevation (m)	Accession no.
GL11	*Quercus oblongata*	Dead stumps	Tara Devi Forest, Shimla	N 31° 05′ 57.2″	1899	PQ312647
				E 077° 06′ 43.7″		
GL13	*Cedrus deodara*	Dead stumps	Summer Hill, Shimla	N 31° 06′ 50.7″	2055	PP843921
				E 077° 08′ 05.7″		
GL17	*Quercus floribunda*	Dead stumps	Himri Forest, Shimla	N 31° 13′ 19.5″	2207	PQ312642
				E 077° 17′ 07.5″		
GL20	*Dalbergia sissoo*	Live tree	FRS Nalagarh	N 31° 2′ 20.956″	286	PQ312685
				E 076° 37′ 26.91″		
GL24	*Quercus semecarpifolia*	Dead tree	Sidpur, Narkanda	N 31° 15.357″	2804	PQ312686
				E 077° 30.152″		

### 2.3. Proximate analysis of the collected samples

AOAC (Association of Official Analytical Chemists) standard procedures were used to establish the approximate composition of *Ganoderma* powder. Moisture content, crude protein, crude fat, ash content, and crude fibre were among the metrics assessed. Crude protein content was determined using the Kjeldahl distillation method, which involves acid digestion to convert organic nitrogen into ammonium ions, followed by alkaline distillation and titration, and mineral content was estimated using ICPOES (Inductively Coupled Plasma Optical Emission Spectroscopy).^12^

### 2.4. Obtention of the extract

The dried powder (1 gram) was extracted in a Soxhlet apparatus for 5–6 hours (35 ± 5 min per cycle) using 70% (v/v) ethanol (100 mL). After extraction, the solutions were filtered through a 0.22 µm membrane to eliminate residual material. The filtrates were concentrated under decreased pressure using a rotary evaporator (Heidolph, Germany) at 40 ± 2 °C to prevent heat destruction of bioactive chemicals. These extracts were used for the phytochemical studies.^13^

### 2.5. Phytochemical characterization of *Ganoderma* samples

#### 2.5.1. Determination of total phenolic content

The total phenolic content of the ethanolic extract of the samples was determined using a modified Folin–Ciocalteu technique. Briefly, 0.5 mL of extract (1 mg mL^−1^) was combined with 2.5 mL of diluted Folin–Ciocalteu reagent (1 : 10, v/v with distilled water). After complete mixing, 2 mL of sodium carbonate solution (75 g L^−1^) was introduced. The reaction mixture was vortexed for 15 seconds before being incubated at 40 °C for 30 min to allow color to develop. A UV-visible spectrophotometer was used to assess absorbance at 765 nm in comparison to a methanol blank.^14^ A calibration curve was prepared using gallic acid in the concentration range of 0.0094–0.15 mg mL^−1^. The total phenolic content was expressed as mg of gallic acid equivalents per gram of dry weight (mg GAE/g dw) and calculated using the following equation:1
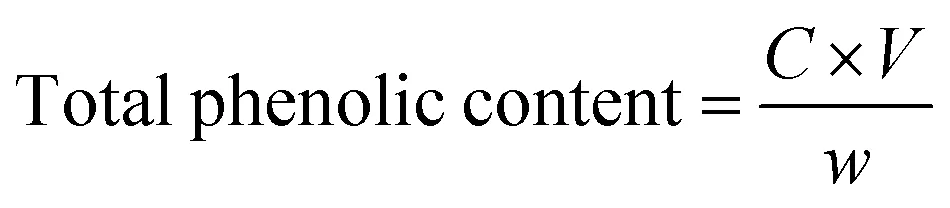
where *C* is the gallic acid concentration obtained from the calibration curve (mg mL^−1^), *V* is the volume of extract used (mL), and *w* is the dry weight of the *G. lucidum* sample in grams.

#### 2.5.2. Determination of terpenoid content

A known quantity (2.5 mg) of *Ganoderma* ethanolic extracts was dissolved in 1 mL of methanol. Transfer 100 µL of the solution to a test tube, then add 150 µL of vanillin-glacial acetic acid reagent (5% w/v) and 500 µL of 70% perchloric acid.^15^ The reaction mixes were produced in triplicate. The tubes were incubated in a water bath at 60 °C for 45 minutes, then rapidly cooled in an ice bath to stop the reaction. After cooling, each tube received 2.25 mL of glacial acetic acid, which was completely mixed in. The absorbance was measured at 548 nm with a UV-visible spectrophotometer against a methanol blank. Ursolic acid was used as the reference standard to construct a calibration curve in methanol over a concentration range of 0.0078–0.5 mg mL^−1^. The total triterpene content was expressed as mg of ursolic acid equivalents per gram of dry weight.

#### 2.5.3. Study of antioxidant activity

The antioxidant activity of *Ganoderma* samples was evaluated using two complementary *in vitro* assays: the 2,2-diphenyl-1-picrylhydrazyl (DPPH) radical scavenging assay and ferric reducing antioxidant power (FRAP) assay.

##### 2.5.3.1. DPPH assay

The antioxidant activity of the extracts was determined using the DPPH radical scavenging activity assay. Briefly, 2.0 mL of a 0.2 mM DPPH solution in methanol was combined with 2.0 mL of the sample at varying concentrations ranging from 0.05 to 0.25 mg mL^−1^.^16^ The mixture was vortexed and incubated in the dark at 30 °C for 15 min. Following incubation, the absorbance was recorded at 517 nm using a spectrophotometer (Thermo Fisher, Evolution 201, UV-visible spectrophotometer). All experiments were performed in triplicate, the percentage of radical scavenging activity was calculated, and ascorbic acid was used as a standard of antioxidant.

Calculation:2
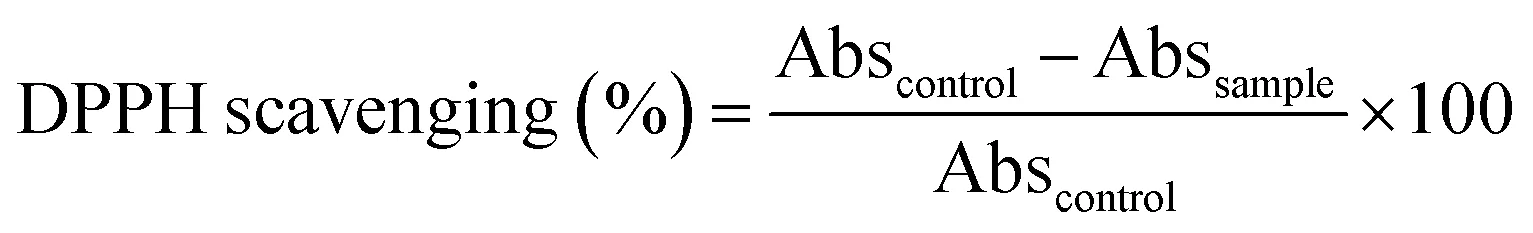
where Abs_control_ represents the absorbance of the DPPH solution with methanol (blank control), Abs_sample_ is the absorbance of the solution containing the DPPH reagent and the sample extract.

##### 2.5.3.2. FRAP assay

The FRAP reagent was freshly prepared by mixing 2.5 mL of TPTZ solution (10 mM TPTZ dissolved in 40 mM HCl), 2.5 mL of 20 mM FeCl_3_ solution, and 25 mL of sodium acetate buffer (300 mM, pH 3.6). The reagent was pre-warmed to 37 °C before use. Mix 3.0 mL of the freshly produced FRAP reagent with 100 µL of sample extract. To complete the reduction of ferric ions, the reaction mixture was incubated for 10 minutes at 37 °C.^17^

After incubation, the absorbance was measured at 593 nm with a UV-visible spectrophotometer. To produce a calibration curve, standard solutions of ferrous sulfate (FeSO_4_) were used under similar circumstances. The antioxidant capacity of the samples was calculated from the FeSO_4_ calibration curve and expressed as µM of FeSO_4_ equivalents per gram of dry weight.

#### 2.5.4. GC-MS analysis

The ethanol extract of the samples was prepared for GC-MS analysis with ethanol at a concentration of 1 mg mL^−1^. GC-MS (GC-Trace1300/GCMS-TSQ-Duo, Thermo Fisher, USA) was used to examine the fraction components, prepared with an auto-sampler (TriPlusRSH) and TG-5MS column (40 m × 0.15 mm × 0.15 µm). The operating conditions of GC-MS were used to screen the extract with minor adjustments. The starting temperature of the process was set at 70 °C and maintained for one minute. Following that, the temperature was raised at a rate of 7 °C per minute until it reached 270 °C and was maintained for 2 minutes. Finally, the temperature was maintained at 270 °C and was sustained for 20 minutes.^18^ The temperature of the transfer line was 250 °C; the carrier gas used was helium and maintained at a constant flow rate of 0.7 mL min^−1^ with the split less injection mode; the component ionization mode was electron impact (70 eV); the ion source temperature was 250 °C; the run time was 50 min; and the mass range was 45–450 *m*/*z*.

### 2.6. Statistical analysis

Proximate parameters and antioxidant capabilities were statistically evaluated in triplicate (*n* = 3) using one-way ANOVA and Tukey's HSD *post-hoc* test (*α* = 0.05). While shared letters denote homogeneous groupings, mean values within the same row with different superscript letters (a–e) are statistically substantially different (*p* < 0.05). OriginLab (OriginLab Corp., USA) and Python (v3.x) were used to perform univariate computations. In order to filter baseline biological noise for mineral profiling and high-throughput untargeted metabolomics, representative composite sample extracts were examined. Auto-scaling (*z*-score) was used to normalize the 179-compound GC-MS dataset. MATLAB (MathWorks, USA) was used to model global PCA, HCA, and correlation heatmaps. Pearson correlation coefficients (*r*) were used to assess inter-element mineral interactions.

## 3. Results and discussion

### 3.1. Collection of the samples

**Fig. 2:**
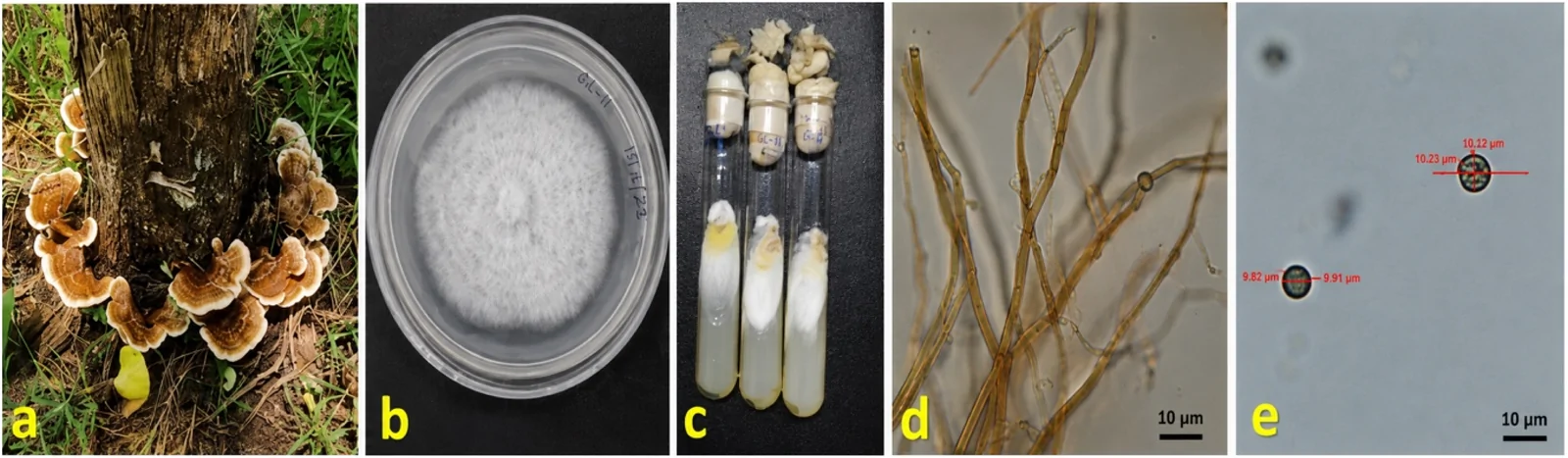
(a) Sporocarps of *Ganoderma*; (b) mycelium; (c) pure culture; (d) hyphae and (e) basidiospores (at scale = 10 µm).

The identified *Ganoderma* isolates showed different macroscopic and microscopic features, validating their taxonomic classification within the genus *Ganoderma* (Table 2). Macroscopically, the majority of isolates (GL11, GL13, GL17, and GL24) had a laccate pileus, whereas GL20 did not, demonstrating obvious morphological heterogeneity among the isolates (Fig. 2). The pileus color ranged from light brown to dark brown, with white edges, and its shape ranged from discoid, fan-shaped, and auriform. Pileus diameters varied from 2.8 to 8.6 cm, indicating variances in growth and developmental phases. All isolates possessed a dark brown stipe, with stipe length varying from 5.6 to 13.2 cm, the longest being observed in GL13. Microscopic examination revealed that the basidiospores of GL11, GL13, GL17, and GL20 were ellipsoidal and yellowish-brown, with size ranges consistent with *Ganoderma*^19^ (Table 3). Variations in basidiospore dimensions were noted among isolates, reflecting intraspecific diversity. The combined macroscopic and microscopic features supported identification of collected samples as *Ganoderma* spp., with characteristics largely consistent with *G. lucidum.*^20^

**Table 2:** Macroscopic characterization of the collected *Ganoderma* samples

Isolates	Pileus				Stipe	
	Texture	Colour	Shape	Diameter (cm)	Colour	Length (cm)
GL11	Laccate	Reddish brown with whitish margins	Discoid	3.5–3.1	Dark brown	5.8
GL13	Laccate	Light brown with whitish margins	Fan shaped	8.6–4.2	Dark brown	13.2
GL17	Laccate	Dark brown, white margins	Auriform	3.4–2.8	Dark brown	5.6
GL20	Non-laccate	Dark brown	Discoid	6.8–5.4	Dark brown	6.0
GL24	Laccate	Dark brown, white margins	Discoid	7.3–5.0	Dark brown	7.9

**Table 3:** Microscopic characterization of the collected *G. lucidum* samples

Isolates	Basidiospores		
	Shape	Colour	Size (µm)
GL11	Ellipsoid	Yellowish-brown	5.61–4.66 × 9.45–7.30
GL13	Ellipsoid	Yellowish-brown	6.56–5.23 × 3.88–10.46
GL17	Ellipsoid	Yellowish-brown	3.49–5.23 × 7.26–4.70
GL20	Ellipsoid	Yellowish-brown	3.79–2.83 × 4.37–7.23
GL24	Ellipsoid	Dark brown	4.71–6.56 × 7.29–4.78

### 3.2. Proximate analysis of the collected samples

The proximate composition of *Ganoderma* isolates is shown in Table 4. The five isolates showed significant variations (*p* ≤ 0.05) in a broad range of nutritional parameters. The observed difference in proximate composition among the isolates demonstrates intraspecific nutritional variety within the *Ganoderma* samples, which may be influenced by ecological variables, host relationship, and physiological age upon collection. Values are expressed as mean ± SD (*n* = 3). Statistical analysis was performed using one-way ANOVA followed by Tukey's HSD *post hoc* test. Different superscript letters indicate significant differences at *p* < 0.05.

**Table 4:** Proximate analysis of five *Ganoderma* samples^a^

Sample	GL11	GL13	GL17	GL20	GL24
Moisture (%)	9.25 ± 0.04^c^	5.85 ± 0.17^e^	15.52 ± 0.23^a^	8.38 ± 0.15^d^	12.46 ± 0.09^b^
Ash (%)	7.05 ± 0.17^b^	3.09 ± 0.08^d^	10.11 ± 0.10^a^	7.11 ± 0.20^b^	6.62 ± 0.16^c^
Fat (%)	6.54 ± 0.21^a^	4.69 ± 0.15^b^	2.33 ± 0.12^d^	3.18 ± 0.14^c^	4.75 ± 0.07^b^
Protein (%)	15.26 ± 0.14^d^	18.49 ± 0.27^a^	16.09 ± 0.08^c^	18.23 ± 0.13^a^	17.14 ± 0.07^b^
Crude fibre (%)	16.19 ± 0.08^b^	16.84 ± 0.17^a^	14.71 ± 0.20^c^	17.17 ± 0.14^a^	13.23 ± 0.20^d^
Carbohydrate (%)	45.69 ± 0.25^b^	51.02 ± 0.60^a^	41.02 ± 0.18^c^	45.91 ± 0.22^b^	45.77 ± 0.27^b^
Energy (kcal)	302.73 ± 1.34^b^	320.27 ± 0.03^a^	250.32 ± 1.72^e^	285.26 ± 0.86^d^	294.44 ± 0.79^c^

a Mean values within the same row accompanied by different superscript letters (a–e) are statistically significantly different *p* ≤ 0.05, while shared letters indicate homogeneous subsets with no significant variance.

The ash content, which indicates the amount of minerals deposited, ranged from 3.09 ± 0.08% (GL13) to 10.11 ± 0.10% (GL17). These findings are consistent with those recently published by Singh *et al.*,^21^ which indicate that the ash percentage falls between 2.5 and 11.0% because of various substrate sources that contain minerals. The crude protein ranged between 15.26 ± 0.14% and 18.49 ± 0.27%, with *G. leucocontextum* samples (GL13 and GL17) showing particularly high levels. According to recent research by Peng *et al.*,^22^*G. leucocontextum* often includes a higher nitrogen concentration than *G. lucidum*. Furthermore, significant crude fiber levels (13.23 ± 0.20–17.17 ± 0.14%) were observed, and such structural characteristics of high crude fiber are common in wild species found at high altitudes due to the production of thick cell walls in stress response.^7^ Although total carbohydrate constituted the major compound (41.02 ± 0.18% to 51.02 ± 0.60%), the extremely low-fat content (2.33 ± 0.12% to 6.54 ± 0.21%) demonstrates that the isolated samples from the Himalayas qualify as a high-fiber and low-fat functional matrix. The energy estimates (250.32 ± 1.72 to 320.27 ± 1.34 kcal per 100 g) are consistent with the metabolic energy requirements of premium medicinal *Ganoderma* species globally.^12^

#### 3.2.1. Multivariate analysis of mineral profiles

**Fig. 3:**
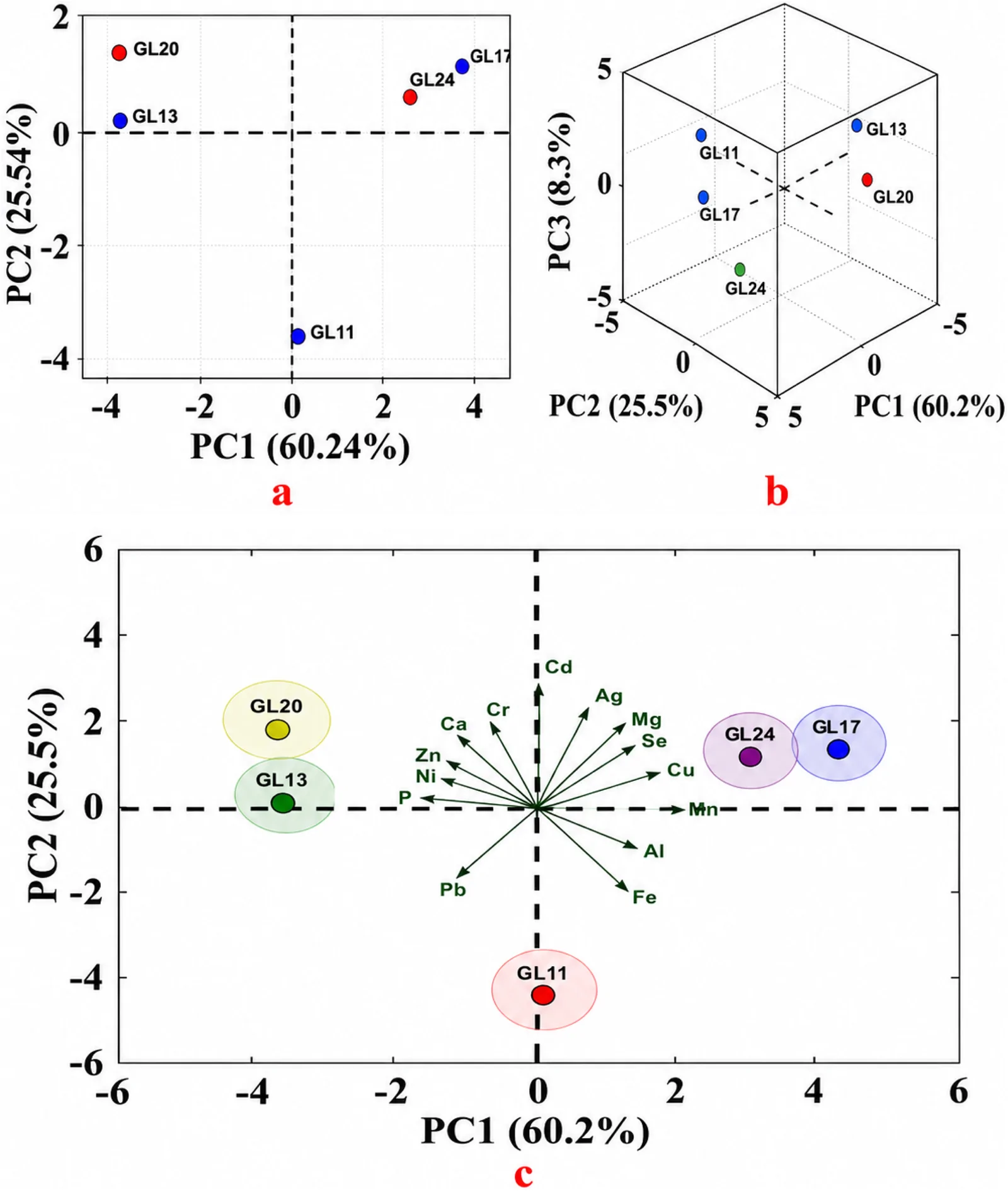
Multivariate statistical analysis of elemental composition in five *Ganoderma* samples. (a) 2D and (b) 3D score plots illustrating the spatial distribution and clustering of samples (c) PCA biplot showing the correlation between samples (scores) and specific mineral variables (loading vectors). Shaded eclipses indicate distinct compositional groups.

The principal component analysis (PCA) was used to investigate the effect of variation in element concentration (Table S1) related to species and host on five samples. The results from Fig. 3a demonstrate that PC1 and PC2 contributed 60.24% and 25.54%, respectively, with the first two PCs contributing 85.78%. The PC3 axis contributed 8.29% of the variation (Fig. 3b). GL17 (3.8178, 1.1374) and GL24 (2.8093, 0.9259) were grouped on the positive PC1 plane, implying comparable elemental compositions. The outliers GL13 (−3.3098, 0.21693) and GL20 (−3.5101, 1.5625) were on the negative PC1 plane, while the outlier GL11 (0.1928, −3.8428) was easily distinguished from the others by its negative PC2 score (Table S2). The high cumulative variance suggests a very structured data set and supports the idea that chemical fingerprints form an accurate low-dimensional description of isolates, as is common in chemometrics of metabolites or minerals produced by fungi.^23^

The score plot for the PCA clearly demonstrated ecological stratification, with the key components being host and elevation. GL17 and GL24 isolates from *Quercus* species hosts (*Q. floribunda* and *Q. semecarpifolia*) at elevations ranging from 2207 to 2804 meters were found to be closely related in the positive PC1 quadrant. The host-specific grouping is consistent with prior research indicating that plant taxonomy plays an important role in the formation of fungal communities.^24^ The separation of GL13 (*Cedrus deodara*) and GL20 (*Dalbergia sissoo*) in the negative PC1 zone indicates that the element composition differed between the two species due to evolutionary differences. This conclusion is congruent with the hypothesis given by Peay *et al.* (2016), who say that changes in host taxonomy are positively correlated with variations in fungal functioning.^25^ GL11 appears to have an odd elemental composition, despite its close relationship to *Q. oblongata*, due to its apparent migration towards the negative PC2 axis. The microenvironmental circumstances and chemical makeup of the wood substrate in degradation are the most likely causes. Previous research has shown that the host has a modest influence when compared to other parameters such as lignocellulose breakdown.^26^

According to the results of the biplot diagram (Fig. 3c), elevation was a factor in determining *Ganoderma* mineral concentration since isolates at high elevation (GL17, GL24) were readily distinguishable from isolates at medium and low elevation. This is consistent with data on the effects of elevation on physiological and mineral content in soil.^27^ The PCA biplot shows that elements K, Ca, Mg, and Sr play major roles in the differentiation of PC1. Increased values for these elements in samples collected from the hills where *Quercus* trees grow indicate that the elements are more readily available to microbes in this environment. This is consistent with ecological stoichiometry concepts since it emphasizes the relationship between microbial elemental composition and nutritional gradients. The geochemical affinity and movement of Ca and Sr elements result in co-loading.^28^

**Fig. 4:**
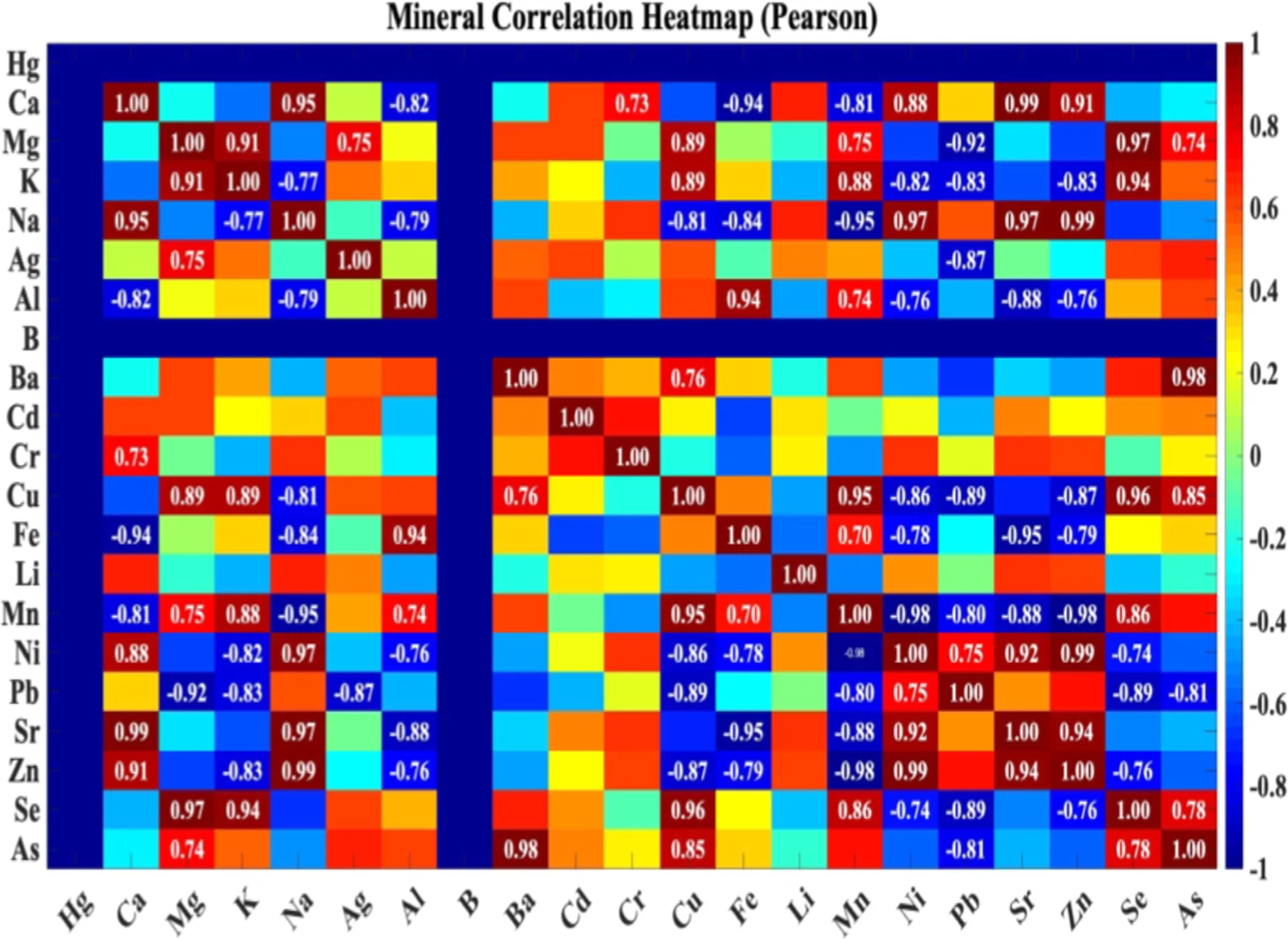
Pearson correlation heatmap displaying the strength of relationships between 21 minerals; values close to 1.00 (red) indicate strong positive correlation, while values close to −1.00 (blue) indicate strong inverse relationships.

A Pearson correlation analysis using a heatmap was performed on the 21 mineral contents in the samples (Fig. 4). The results revealed strong positive correlations (*R* > 0.90) between elements like Ca–Sr (*R* = 0.99), Na–Zn (*R* = 0.99), and Mg–Se (*R* = 0.97), indicating similar geochemical sources or transportation processes. Conversely, negative correlations (*R* < 0.85) between elements like Mn–Na (*R* = −0.95) and Fe–Ca (*R* = −0.94) indicate competition. The research revealed very strong positive correlations (*R* > 0.90), indicating a close connection between the behavior of elements such as Ca–Sr (*R* = 0.99), Na–Zn (*R* = 0.99), and Mg–Se (*R* = 0.97), supporting similar geological sources or transportation pathways. Negative correlations (*R* < 0.85) for Mn–Na (*R* = −0.95) and Fe–Ca (*R* = −0.94) indicate competitive binding and interaction during membrane transport. The robust Ca–Sr and Na–Zn networks merged to produce a separate Ca–Sr–Na–Zn cluster, which corresponded to isolates collected from *Quercus* substrates at higher elevations. Concurrently, a distinct Cu–Cr–Mn–Ni cluster formed, revealing localized trace metal co-accumulation. These non-random elemental profiles show that *Ganoderma* mineral composition is not just an environmental artifact but the result of coordinated absorption systems influenced by host substrate chemistry and significant altitude gradients.

### 3.3. Chemometric analysis of *Ganoderma* antioxidant potential

**Fig. 5:**
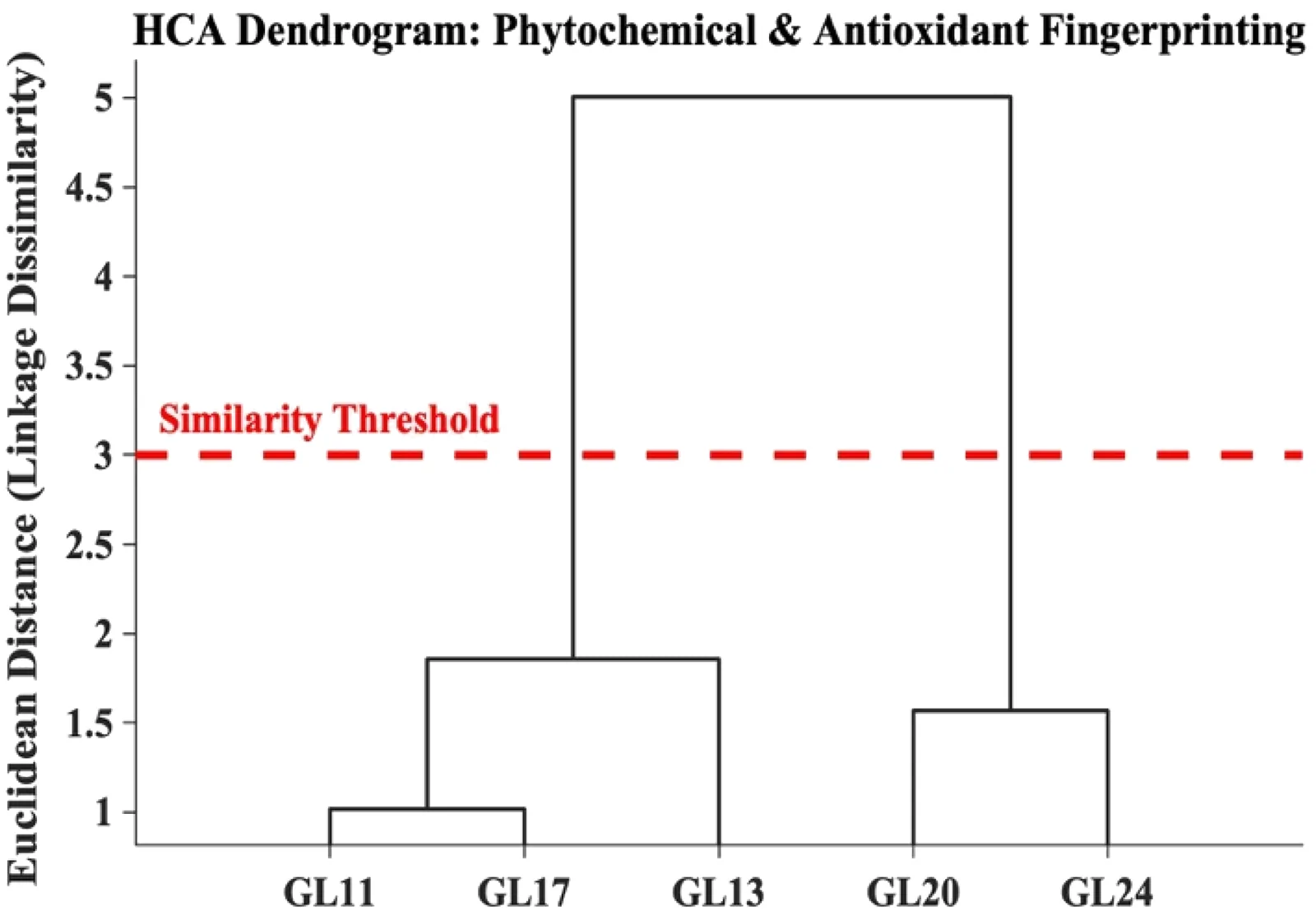
Hierarchical clustering analysis (HCA) dendrogram of five *Ganoderma* isolates based on phytochemical and antioxidant fingerprinting.

The pairwise Euclidean distance matrix was used in a chemometric multivariate hierarchical clustering analysis (HCA) to investigate the connection between isolate identification and antioxidant capacities (Table S3). The five isolates were divided into two main clusters using a similarity threshold of 3.0 based on phenon line optimization: Cluster I, which included *G. leucocontextum* strains (GL11, GL13, and GL17), and Cluster II, which included *G. lucidum* strains (GL20 and GL24). GL11 and GL17 were shown to be the most chemically similar pair within Cluster I with the shortest spatial distance (*D* = 1.018) by the matrix, and a significant, species-specific phenotypic segmentation is confirmed by the huge linkage distances above 3.0 between the two main groups (Fig. 5).

**Fig. 6:**
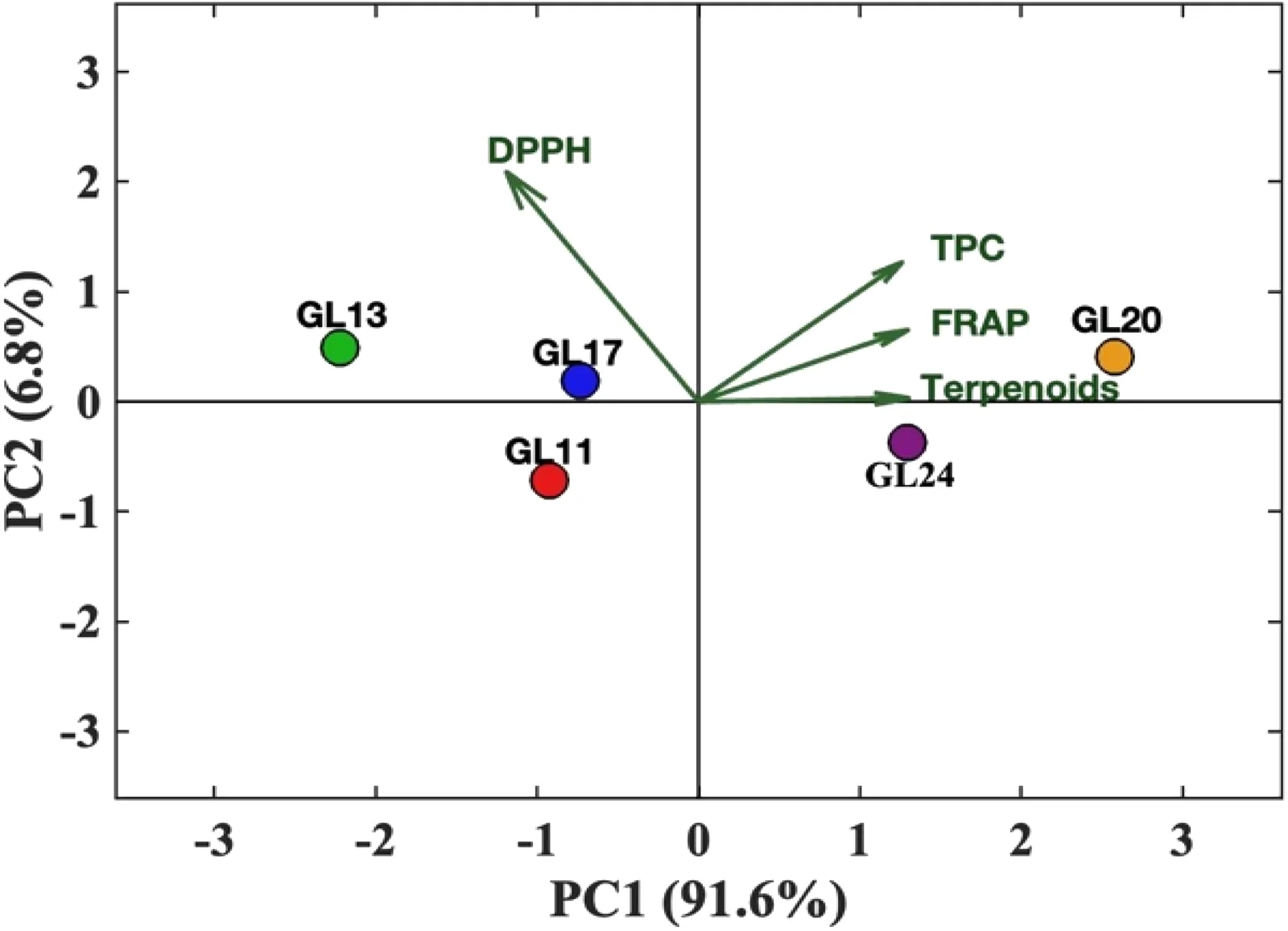
PCA biplot of five *Ganoderma* isolates showing relationships with antioxidant parameters.

PCA revealed more information on the chemical cause for this separation (Fig. 6). PC1 captured 91.6% of the variance, while PC2 accounted for 6.8%. The PCA figure shows that *G. lucidum* strains (GL20, GL24) are associated with TPC, terpenoids, and FRAP values, while *G. leucocontextum* strains (GL11, GL13, GL17) correlated more with DPPH scavenging activity.

The total chemometric data clearly reveal that the antioxidant activities of *Ganoderma* isolates are impacted more by their species identity than by their isolate identity. The clustering of *G. leucocontextum* with DPPH suggests that the property is based on radical scavenging, whereas the clustering of *G. lucidum* with phenolics, terpenoids, and FRAP implies reducing capability. This finding is consistent with several prior studies on *Ganoderma*, in which *G. lucidum* isolates are known to be high in triterpenoids and have ferric ion reduction capacity.^29,30^

### 3.4. GC-MS multivariate analysis

PCA was applied to evaluate the chemical profiles of five *Ganoderma* isolates based on 179 metabolites, resulting in a data matrix of 5 × 179. To ensure all compounds contributed equally to the model regardless of their absolute concentration, auto-scaling (a combination of mean centering and unit variance scaling) was employed as the preprocessing technique. This transformation standardizes the dataset such that each variable has a mean of zero and a variance of one. In the context of complex GC-MS metabolomics, multivariate techniques offer a significant advantage over simple visual observation of raw peak areas, as they allow for the simultaneous evaluation of the entire metabolic fingerprint. PCA was utilized here to uncover behavioural patterns and latent chemical trends across the isolates that are not immediately obvious in standard data tables. By reducing the dimensionality of the 179-compound dataset, this chemometric tool reveals relevant information regarding isolate-specific biomarkers and overarching metabolic similarities.^31^

PCA was performed on the GC-MS metabolomic profile data set with MATLAB to reduce its dimension and extract any latent tendencies in the chemical structure. To standardize the loading vector values (*L*), the eigenvector values (*V*) were scaled by the square root of the accompanying eigenvalue (*γ*), as given in eqn (3). Such standardization allows us to assess the influence of each metabolite in terms of correlation along the primary axis components, as is common practice in metabolomics.

3 *L*_std_ = *V*√*γ*

**Fig. 7:**
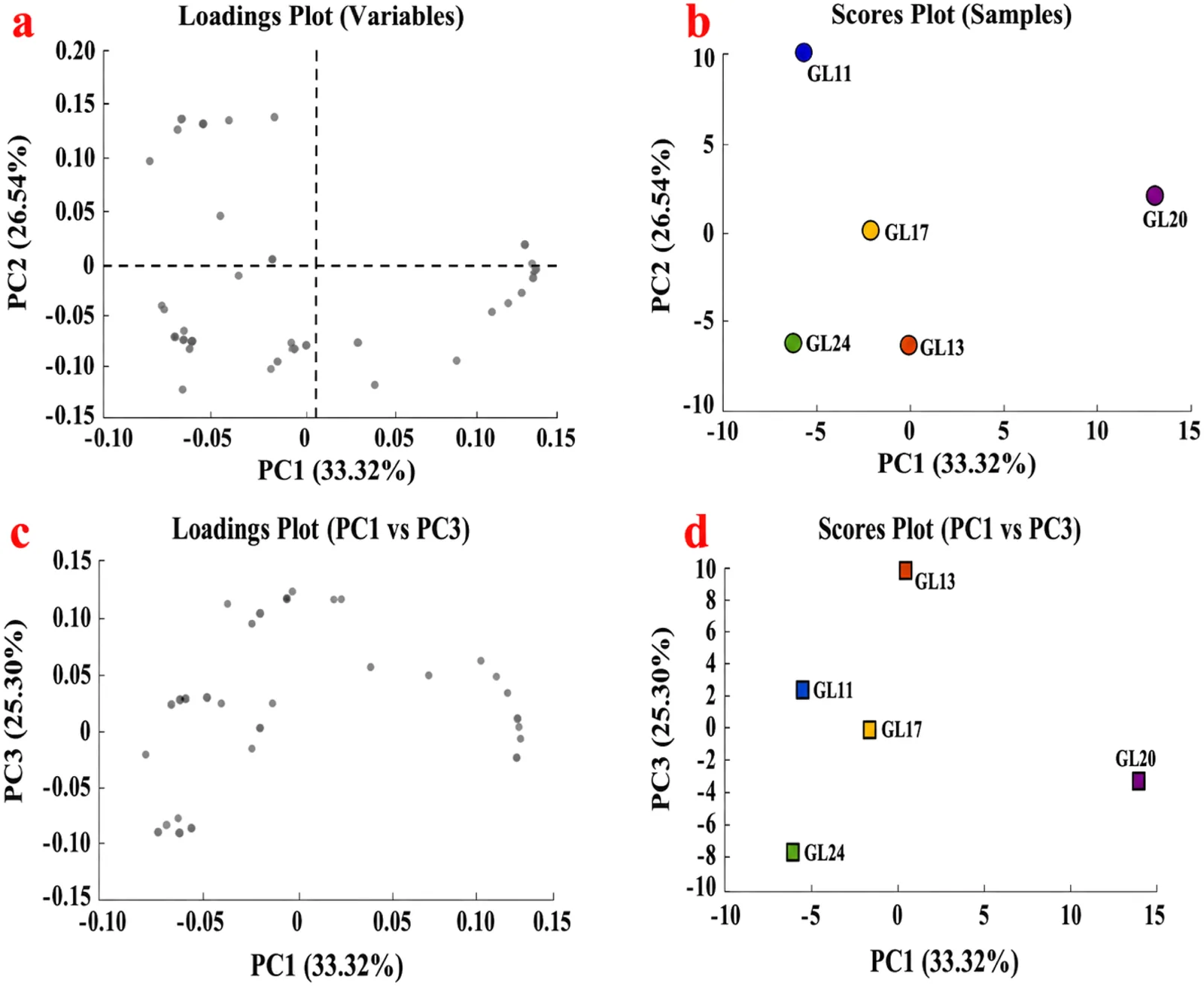
(a) Primary chemometric landscape (PC1 *vs.* PC2). (Left) Loadings plot representing the distribution of the 179-compound metabolic fingerprint. (b) (Right) Scores plot illustrating the spatial segregation of isolates (GL11: blue, GL13: orange, GL17: yellow, GL20: purple, GL24: green). Combined variance: 59.86%. (c) Secondary chemometric resolution (PC1 *vs.* PC3). (Left) Loadings plot demonstrating the shift in variance toward specialized metabolites. (d) (Right) Scores plot providing orthogonal resolution of GL13 and GL24. Cumulative variance: 85.16%.

PCA explained 85.16% of the total variation using the first three main components, demonstrating the robustness of the statistical method. PCA's ability to extract information from chemical profiles has also been proven in various research on fungal metabolomics. In the PC1 and PC2 biplots (Fig. 7a and b), 59.86% of the variation was explained. The GL11 isolate separated along the positive PC2, affected by lipid-derived esters and polyols such as the ethyl ester of hexadecanoic acid (marker 2, |*L*| = 0.9900) and ethanethiol derivative (marker 5, |*L*| = 0.9855). The clustering of octadecenoic acid derivatives and diglycerol (markers 1, 4, 8) indicates that GL11 has a distinct carbon partitioning system as compared to the common lipids of GL20 and GL24 (Table S5). Because of their primary metabolic variety, lipid esters have long been recognized as distinguishing markers of *Ganoderma* metabolomics.^32^

**Fig. 8:**
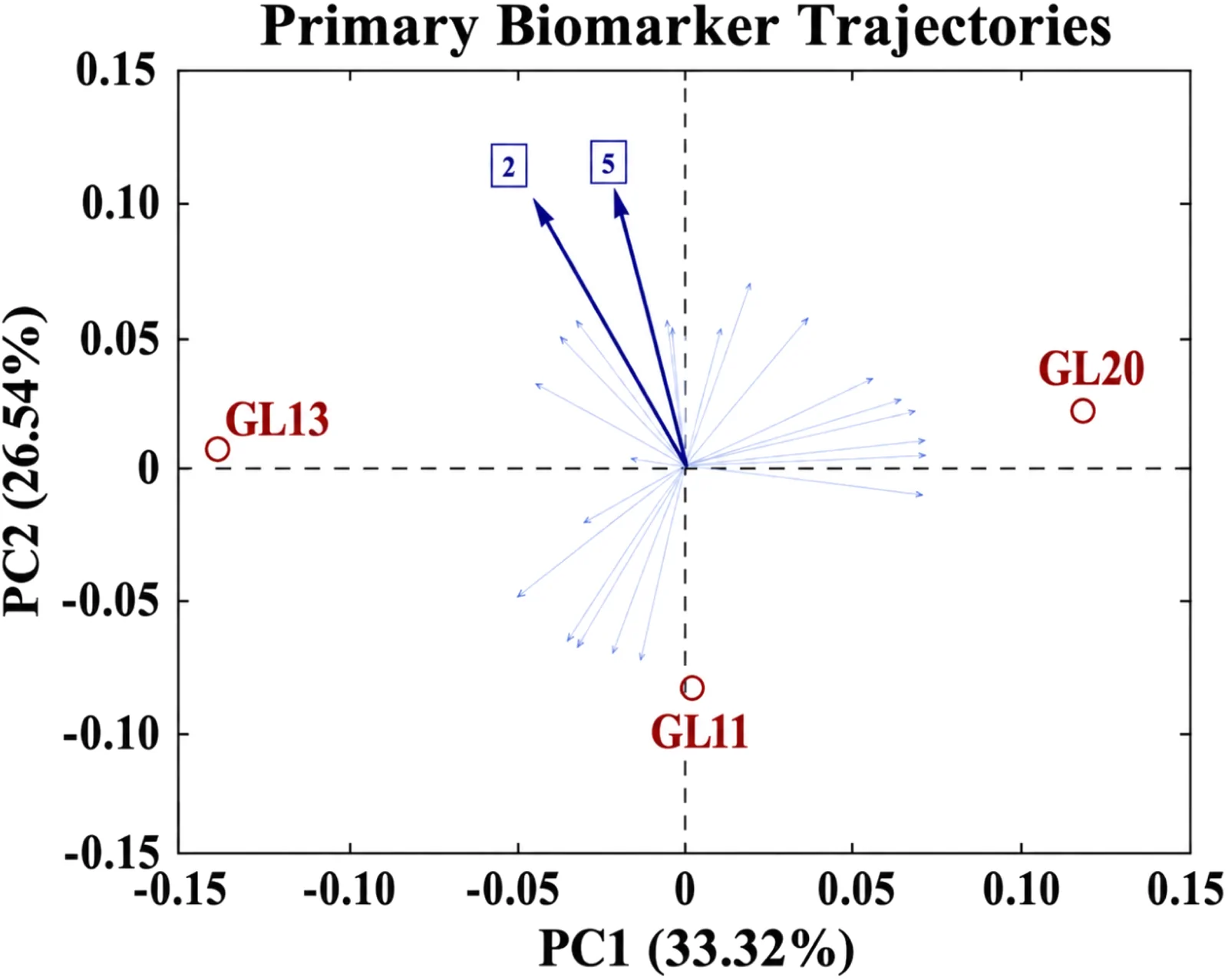
PC1 *vs.* PC2 biplot: primary biomarker trajectories. Integrated projection of isolate scores (red circles) and prioritized metabolic vectors (blue). The highlighted trajectories correspond to hexadecanoic acid, ethyl ester (marker 2) and ethanethiol, 2-(diethylboryloxy)-(marker 5).

**Fig. 9:**
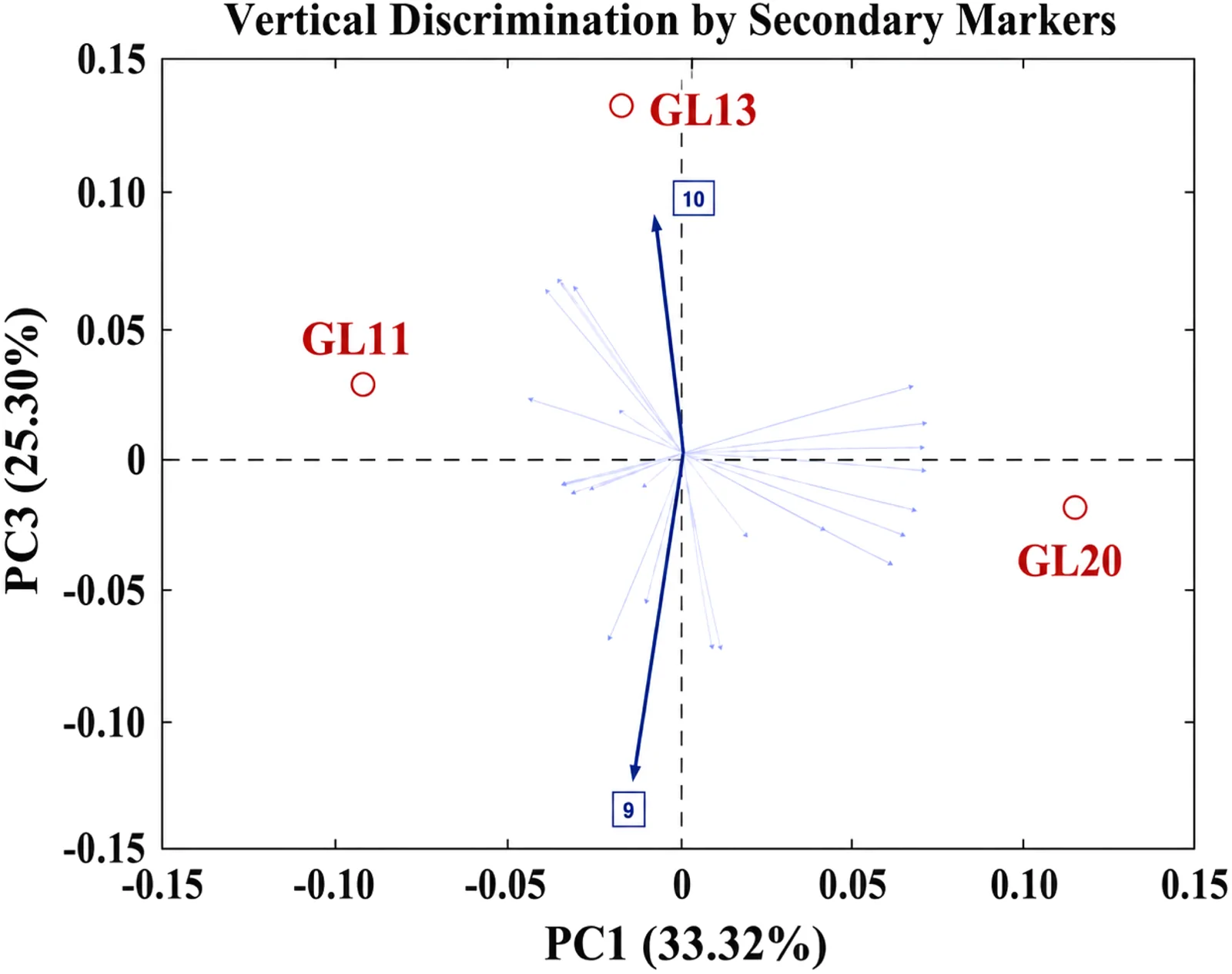
PC1 *vs.* PC3 biplot: integrated projection of isolate scores (red circles) and prioritized metabolic vectors (blue). Illustrating vertical isolate discrimination (25.30% variance). The separation along PC3 (25.30%) is primarily driven by marker 9 (1*H*-indene, 1-methylene-) and marker 10 [2,6-dimethyl-*N*-[3-(trimethylsilyl)-1,3-thiazinan-2-ylidene] aniline].

To address the issue of GL13 and GL17 being near in PC1/PC2 space, another orthogonal projection, this time between PC1 and PC3 (Fig. 7c and d), was investigated, with an additional 25.30% of variability considered. As a result of this projection, it became obvious that GL13 was distinguished from GL24 by the existence of specific, unique secondary metabolites but no primary lipids. Aromatic and bicyclic molecules, including 1*H*-indene, 1-methylene (marker 9), and aniline (marker 10), have high standardized values of |*L*| ≈ 0.9854 and were utilized as high-resolution chemical barcodes. Fig. 8 and 9 depict the integration of the isolates (red circles) and biomarkers (blue). Markers 2 and 5 play an important role in explaining the phenotypic distinction for GL11 in the PC1 *vs.* PC2 plot. Similarly, the PC1 *vs.* PC3 figure uses two aromatic markers, 9 and 10, to distinguish between GL13 and GL24. This synchronization of the isolates with the trajectory of biomarkers makes the latter discriminators more trustworthy, similar to PCA-based biomarker selection techniques.

### 3.5. Integrated multi-block landscape

**Fig. 10:**
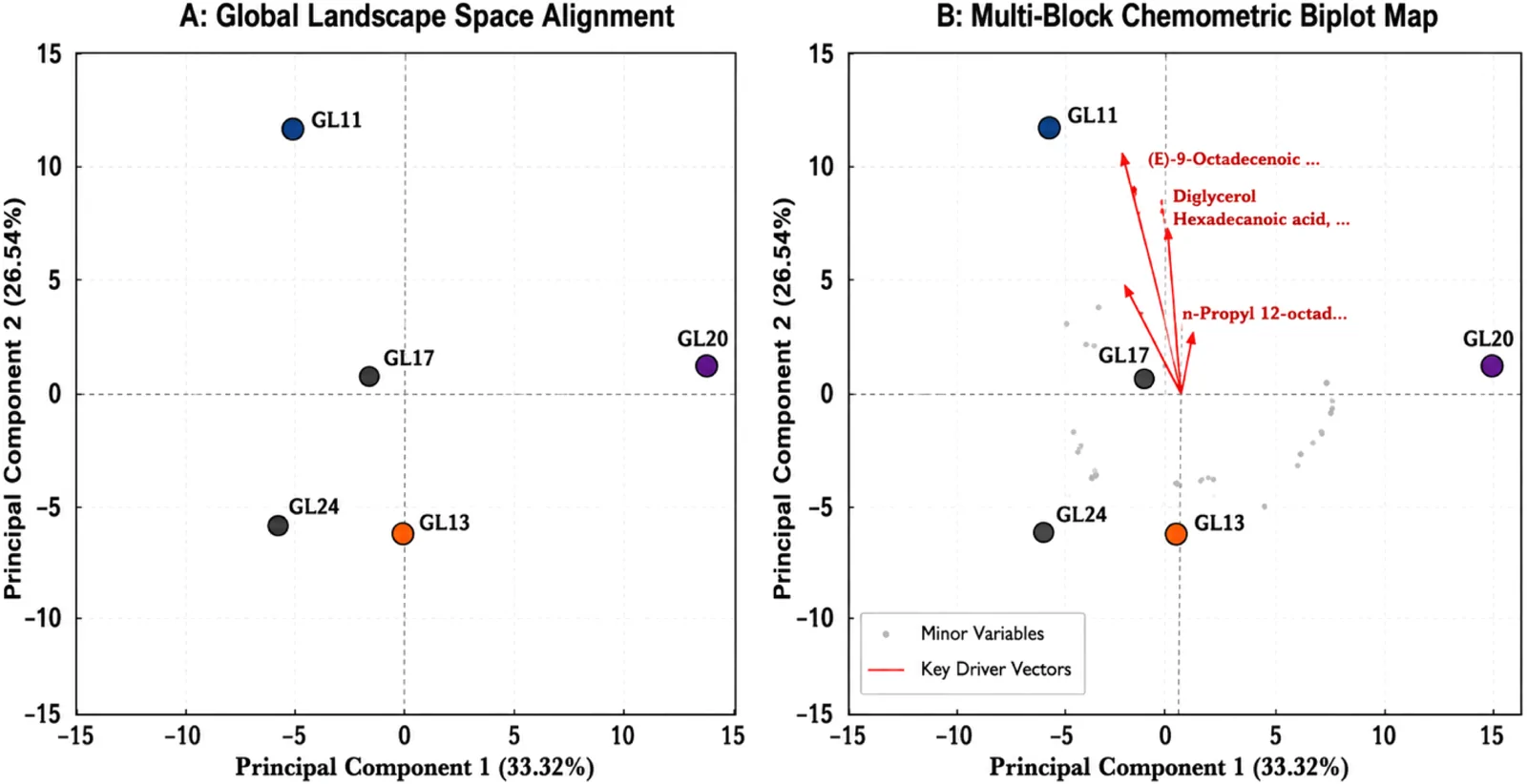
Integrated (A) global landscape space alignment (B) multi-block chemometric biplot map.

A global multi-block PCA, a dimensionality-reduction technique that summarizes major sources of variation, was carried out on a standardized 5 × 211 matrix in order to combine the proximate composition, mineral profile, targeted antioxidant dataset, and 179 untargeted GC-MS variables into a single chemotaxonomic framework (Fig. 10). Unit-variance autoscaling was used before the model and resulted in PC1 and PC2 explaining 59.86% of the overall variance, divided into 33.32% and 26.54%, respectively. The five Himalayan *Ganoderma* isolates displayed distinct chemotype-level differentiation in the global ordination space. Instead of integrating with the other isolates, GL11 remained an isolated chemotype within the integrated dataset, as seen by its separate location in the upper-left quadrant along the positive PC2 axis. Further analysis of the loading structure revealed that a coordinated multi-block signature, not a single anomalous variable, was responsible for the separation of GL11. Crude fat, elevated iron and aluminum, and unique volatile markers like (*E*)-9-octadecenoic acid ethyl ester, 10-octadecenoic acid methyl ester, diglycerol, and hexadecanoic acid ethyl ester, all of which projected toward the GL11-associated region of the biplot, were the strongest contributors. Low-contributing variables were suppressed into faint background points for visual clarity, preserving the interpretability of the dominating discriminant vectors without eliminating the larger chemical context. All things considered, the integrated multi-block PCA offers a coherent chemometric baseline that contextualizes the finer-scale separations shown in the individual biplots and supports the isolate-specific classification of Himalayan *Ganoderma*. Such integrative analysis is in line with well-established multi-block chemometric procedures that preserve interpretable block contributions while extracting global components from diverse analytical blocks using concatenated or consensus PCA.^33^ Untargeted volatile metabolomics can resolve species boundaries and discover flag molecules that cause separation in multivariate space, according to similar chemotaxonomic investigations.^34^ The interpretation of GL11 as a unique chemotype rather than a straightforward analytical outlier demonstrates that standardized, merged GC-MS datasets may recover species-specific VOC signatures.

## 4. Conclusion

The current study examined the nutritional, mineral, antioxidant, and metabolomics characteristics of five *Ganoderma* isolates from the Himalayas. The ANOVA test on proximate analysis data revealed intraspecies variation, with GL13 having the highest protein and carbohydrate content, GL17 having the highest ash content, and GL20 having the highest fibre content, indicating that *Ganoderma* species have diverse nutritional value, even within a single genus. The PCA and Pearson correlation on mineral profile explained 85.78% of the variance and revealed a substantial positive association between Ca–Sr and Na–Zn, as well as a negative correlation between Mn–Na and Fe–Ca, with *Quercus*-assisted high-altitude isolates clustering together in their own groups. GC-MS metabolomics identified 179 metabolites, with PCA accounting for 85.16% of the variation. Ester lipids caused significant separation, while aromatic compounds formed finer chemical fingerprints. Biomarkers with medicinal applications include hexadecanoic acid ethyl ester and octadecenoic acid compounds, which have anti-inflammatory, antioxidant, and hypocholesterolemic properties. Polyols (mannitol, sorbitol) serve an important function in osmoregulation and stability and are often used in medication preparations. Phenolics have strong antioxidant and anticancer properties, whereas terpenoids are renowned for their immunomodulating, hepatoprotective, and antitumor effects. Aromatic molecules such as 1*H*-indene, 1-methylene-, and an aniline compound have antibacterial and anti-inflammatory properties and serve as finer chemotaxonomic indicators. In conclusion, a metabolic hierarchy where the host tree species and altitude are thought to be the main ecological factors influencing *Ganoderma's* biochemical phenotypic profile and medicinal significance was created by combining methods like ANOVA, PCA, HCA, and Pearson's correlation. This combined multivariate study confirms the metabolomic characterisation of Himalayan *Ganoderma* species by demonstrating their metabolic variability.

## Author contributions

Sonali Khanal: writing – original draft, visualization, validation, software, resources, methodology, investigation, formal analysis, data curation, conceptualization. Pankaj Kumar: writing – original draft, software, methodology. Purnima Sharma: resources and data curation. Vinay Chauhan: software, investigation, formal analysis; Rachna Verma: software, investigation, formal analysis, data curation; Ashwani Tapwal: visualization, validation, and supervision. Dinesh Kumar: data curation, conceptualization, and methodology. Vinod Kumar: software, resources, and data curation.

## Conflicts of interest

The authors declare that they have no known competing financial interests or personal relationships that could have appeared to influence the work reported in this paper.

## Supplementary Material

RA-016-D6RA04253H-s001

## Data Availability

The datasets generated and/or analyzed during the current study are available from the corresponding author upon reasonable request. All raw and processed data supporting the findings, including proximate composition, mineral profiling, antioxidant assays, and GC-MS metabolomic outputs, have been archived and can be shared for academic purposes. Supplementary information (SI): summarizes data of mineral composition, PCA score plot, pairwise Euclidean distance matrix for the antioxidant profiling, phytochemical profiling of the five isolates, and standardized metabolic driver prioritization (top 10) in tabular form. See DOI: https://doi.org/10.1039/d6ra04253h.
