# Supplementary material for: Multivariate chemometric profiling of Himalayan Ganoderma isolates: linking host and altitude to nutritional, antioxidant, and metabolomic diversity
Source: RSC Adv. 2026 Jul 27;16(38):42996–3006. doi: 10.1039/d6ra04253h (PMC13404364; doi:10.1039/d6ra04253h)
Supplement: Supplementary file 1 [file RA-016-D6RA04253H-s001.pdf]

Supplementary Information (SI) for RSC Advances.
This journal is © The Royal Society of Chemistry 2026

Multivariate chemometric profiling of Himalayan *Ganoderma* isolates: linking host and altitude to nutritional, antioxidant, and metabolomic diversity

Sonali Khanal[1], Pankaj Kumar[2], Purnima Sharma[3], Vinay Chauhan[2], Rachna Verma[4,5], Ashwani Tapwal[3], Dinesh Kumar[1,5*], Vinod Kumar[6*]

[1]School of Bioengineering and Food Technology, Shoolini University of Biotechnology and Management Sciences, Solan 173229, India

[2]School of Advanced Chemical Sciences, Shoolini University, Solan 173229, India

[3]ICFRE-Himalayan Forest Research Institute, Shimla, 171013

[4]School of Biological and Environmental Science, Shoolini University of Biotechnology and Management Sciences, Solan 173229, India

[5]Centre of Advanced Innovation Technologies, VSB – Technical University of Ostrava, 708 00, Ostrava-Poruba, Czech Republic

[6]Magan Centre for Applied Mycology, Cranfield University, Cranfield MK43 0AL, United Kingdom

***Joint corresponding Authors:**

Dr. Dinesh Kumar

***Email:** dineshkumar@shooliniuniversity.com*

Dr. Vinod Kumar

***Email:** vinod.kumar@cranfield.ac.uk*

Table S1: Mineral composition

Parameters	**GL11(as mg/kg) on DW**	**GL13(as mg/kg) on DW**	**GL17(as mg/kg) on DW**	**GL20(as mg/kg) on DW**	**GL24(as mg/kg) on DW**
Mercury	0.01	0.01	0.01	0.01	0.01
Calcium	312.07	2175.21	747.81	2572.39	943.98
Magnesium	130.08	239.42	551.45	270.51	667.50
Potassium	2091.12	1324.35	4870.52	708.60	7709.36
Sodium	213.02	1457.31	0.1	1796.64	56.90
Silver	0.01	0.01	0.1	0.1	0.1
Aluminium	340.64	69.46	456.30	86.93	170.08
Boron	0.1	0.1	0.1	0.1	0.1
Barium	1.88	2.87	6.79	2.76	3.28
Cadmium	0.09	0.1	0.1	0.1	0.1
Chromium	3.61	6.47	5.82	5.80	4.06
Copper	2.21	1.19	9.35	0.51	8.07
Iron	389.79	52.19	345.73	41.51	176.94

Lithium	0.01	0.01	0.05	0.88	0.1
Manganese	9.52	1.65	16.99	0.97	15.96
Nickel	0.86	3.23	0.1	2.83	0.1
Lead	0.77	0.90	0.1	0.53	0.1
Strontium	0.67	8.16	0.75	9.02	2.65
Zinc	3.77	14.19	0.1	15.55	0.1
Selenium	0.1	1.23	6.33	0.1	7.14
Arsenic	1.46	2.56	10.56	3.23.	5.22

DW: dry weight

Table S2: PCA score plot

PC	PC1	PC2	PC3	PC4
**GL11**	0.1928	-3.8428	-0.0619	0.4122
**GL13**	-3.3098	0.2169	0.6093	-1.5010
**GL17**	3.8178	1.1374	1.5850	0.2705
**GL20**	-3.5101	1.5625	-0.3114	1.3260
**GL24**	2.8093	0.9259	-1.8210	-0.5076

Table S3: Pairwise Euclidean Distance Matrix for the antioxidant profiling

GL11	0	1.787	1.018	3.6764	2.2973
GL13	1.787	0	1.5804	4.8079	3.6322
GL17	1.018	1.5804	0	3.343	2.129
GL20	3.6764	4.8079	3.343	0	1.5691
GL24	2.2973	3.6322	2.129	1.5691	0

Table S4: Phytochemical profiling of the five isolates

Sample	TPC (mg GAE/g)	Terpenoids (mg UA/g)	DPPH (IC50)	FRAP (µmol/g)
**GL11**	1.46 ± 0.01	3.14 ± 0.005	229.55 ± 1.04	91.90 ± 0.002
**GL13**	1.31 ± 0.01	2.37 ± 0.005	279.59 ± 0.37	76.66 ± 0.001
**GL17**	1.78 ± 0.01	3.6 ± 0.003	252.36 ± 5.43	111.42 ± 0.003
**GL20**	4.54 ± 0.04	6.36 ± 0.01	208.09 ± 13.23	162.38 ± 0.02
**GL24**	2.83 ± 0.02	5.82 ± 0.009	211.28 ± 3.35	133.33 ± 0.006

Table S5: Standardized metabolic driver prioritization (top 10)

**Marker ID**	Name	**Primary Mag. (1-2)**	**Secondary Mag. (1-3)**
1	(E)-9-Octadecenoic acid ethyl ester	**0.6922**	**0.8673**
2	Hexadecanoic acid, ethyl ester	**0.9900**	**0.4530**
3	Hexadecanoic acid, methyl ester	**0.5224**	**0.8508**
4	Diglycerol	**0.6907**	**0.8669**
5	Ethanethiol, 2-(diethylboryloxy)-	**0.9855**	**0.4463**
6	Propane, 2-fluoro-2-methyl-	**0.9616**	**0.9854**
7	1-Hexadecanol, 2-methyl-	**0.9616**	**0.9854**
8	10-Octadecenoic acid, methyl ester	**0.9616**	**0.9854**
9	1H-Indene, 1-methylene-	**0.9616**	**0.9854**
10	2,6-Dimethyl-N-[3-(trimethylsilyl)-1,3-thiazinan-2-ylidene]aniline	**0.9616**	**0.9854**
